# Extreme retinal remodeling triggered by light damage: implications for age related macular degeneration

**Published:** 2008-04-25

**Authors:** Robert E. Marc, B.W. Jones, C.B. Watt, F. Vazquez-Chona, D.K. Vaughan, D.T. Organisciak

**Affiliations:** 1 Ophthalmology, University of Utah, John A. Moran Eye Center, Salt Lake City, UT; 2 Department of Biology, University of Wisconsin/Oshkosh, Oshkosh, WI; 3 Petticrew Research Laboratory, Department of Biochemistry and Molecular Biology, Wright State University, Dayton, OH.

## Abstract

**Purpose:**

Our objective was to comprehensively assess the nature and chronology of neural remodeling in retinal degenerations triggered by light-induced retinal damage (LIRD) in adult albino rodents. Our primary hypothesis is that all complete photoreceptor degenerations devolve to extensive remodeling. An hypothesis emergent from data analysis is that the LIRD model closely mimics late-stage atrophic age relared macular degeneration (AMD).

**Methods:**

Sprague-Dawley (SD) rats received intense light exposures of varied durations and survival times ranging from 0 to 240 days. Remodeling was visualized by computational molecular phenotyping (CMP) of a small molecule library: 4-aminobutyrate (γ), arginine (R), aspartate (D), glutamate (E), glutamine (Q), glutathione (J), glycine (G), and taurine (τ). This library was augmented by probes for key proteins such as rod opsin, cone opsin and cellular retinal binding protein (CRALBP). Quantitative CMP was used to profile 160 eyes from 86 animals in over 6,000 sections.

**Results:**

The onset of remodeling in LIRD retinas is rapid, with immediate signs of metabolic stress in photoreceptors, the retinal pigmented epithelium (RPE), the choriocapillaris, and Müller cells. In particular, anomalous elevated aspartate levels appear to be an early stress marker in photoreceptors. After the stress phase, LIRD progresses to focal photoreceptor degeneration within 14 days and extensive remodeling by 60 days. RPE and choriocapillaris losses parallel Müller cell distal seal formation, with progressive neuronal migration, microneuroma evolution, fluid channel formation, and slow neuronal death. The remaining retina in advanced light damage can be classified as survivor, light damage (LD), or decimated zones where massive Müller cell and neuronal emigration into the choroid leaves a retina depleted of neurons and Müller cells. These zones and their transitions closely resemble human geographic atrophy. Across these zones, Müller cells manifest extreme changes in the definitive Müller cell τQE signature, as well as CRALBP and arginine signals.

**Conclusions:**

LIRD retinas manifest remodeling patterns of genetic retinal degeneration models, but involve no developmental complexities, and are ultimately more aggressive, devastating the remaining neural retina. The decimation of the neural retina via cell emigration through the perforated retina-choroid interface is a serious denouement. If focal remodeling in LIRD accurately profiles late stage atrophic age-related macular degenerations, it augurs poorly for simple molecular interventions. Indeed, the LIRD profile in the SD rat manifests more similarities to advanced human atrophic AMD than most genetically or immunologically induced murine models of AMD.

## Introduction

In retinal degenerative diseases, photoreceptor death is only the beginning of progressive neural remodeling and reprogramming resulting in cellular death and neural circuit remodeling [1]. This compromises transplant [2] and bionic vision restoration strategies [3,4] and contracts the time frame for gene therapies [5-8]. Remodeling is generic to a range of genetically triggered diseases such as retinitis pigmentosa (RP) and RP-like degenerations in animal models [1,5]. However, the variability in the kinetics of disease onset across photoreceptor degeneration types [1], the varied degrees of cone survival [5], the slow emergence of remodeling, and the overlap between retinal development and expression of gene defects [9] have all been impediments to understanding remodeling chronology and spatial patterns, both of which are key elements in any attempt to design interventions in retinal disease. Thus we explored the onset, nature and scope of remodeling in the well established albino rat light-induced retinal damage (LIRD) model: a system in which we could ensure adult onset and coherent timing of photoreceptor stress.

The ability of prolonged actinic light to initiate delayed regional rod and cell cone death in albino rodents [10] clearly involves multiple components, including photoreceptors via rhodopsin bleaching, the RPE via retinoid processing and generation of mobile oxidative species, and the retinal network that controls the circadian state of the RPE [10]. LIRD severity is graded with light duration, intensity, and circadian phase, often being most pronounced in dorsal (superior) retina [11,12]. The mechanism of regional LIRD remains unclear.

Is exploring this model merely an academic exercise? Despite the fact that the LIRD rodent model is popular as a neuroprotection assay system for oxidative stress in the outer retina and at the photoreceptor-RPE interface, its relevance to human retinal disease and RP-like diseases in particular can be questioned. As we shall demonstrate, however, the LIRD model time-compresses the essential features of human atrophic AMD and, unique among animal models, involves a concise and reproducible breakdown of the RPE-Bruch’s membrane-choriocapillaris complex. Further, as emerging genetic evidence places the potential molecular initiators of the complex of AMD-like diseases at the basal face of the RPE [13-19], and as epidemiologic and nutritional data implicate free radical regulation capacity as a risk factor [20], the rodent LIRD model deserves renewed exploration, especially in terms of cellular movement through the largely cell-impermeant blood-retina interface.

Typical remodeling occurs in three phases: In phase 1, photoreceptor stress is evidenced by photoreceptor outer segment truncation, genotype-dependent opsin delocalization and changes in synaptic architecture, often leading to disconnection of bipolar cells before photoreceptors die [21]. In phase 2, the outer nuclear layer is progressively dismantled via photoreceptor death and active removal of debris, with the likely participation of microglia [22-24]. At the end of phase 2, the network of Müller cell processes that comprise the outer nuclear layer scaffolding collapses into a dense seal similar but not identical to a classical astrocytic scar [25] entombing the neural retina [1]. In classic photoreceptor degenerations, phase 2 also completes the process of bipolar cell dendrite retraction and elimination of all bipolar cell ionotropic (iGluR) [5] and metabotropic mGluR6 glutamate-gated currents [26]. Phase 3 remodeling is heralded by the erratic evolution of new processes from all types of remaining neurons into novel synaptic tufts (microneuromas) that form outside of the normal lamination of the inner plexiform layer, as well as new neurite fascicles that may run for great distances within the neural retina (>0.1 mm). Bidirectional migration of neurons across the layers of the retina also occurs in columns often near hypertrophic Müller cells [1].

By carefully tracking the chronology of remodeling in the Sprague-Dawley rat after intense light exposure, we show that LIRD retinas show the same spectrum of phase 3 remodeling events as other degeneration models and more. Importantly, the loss of the RPE-choriocapillaris complex removes barriers to aggressive Müller cell and neuronal migration and, within 60–120 days after light exposure, focal eruptions of all cell types occur, resulting first in small and ultimately massive streams of cellular emigration from the remnant neural retina, leaving behind a husk of decimated retina. The LIRD retina is the first known retinal disease model where mature neurons transform into fusiform motile cells and leave the nervous system, migrating into other tissues. This predicts that the disruption of the blood-retina interface in AMD, and atrophic AMD in particular, may enable focal emigration of survivor retinal neurons.

## Methods

### Animals

Over 160 retinas from 86 Sprague-Dawley rats (22 controls, 64 LIRD) were analyzed by CMP [1,27,28] using light exposures of varied duration, pre-adaptation states, circadian phases, and survival times (see Table 1). All animal experiments were conducted according to the ARVO Statement for the Use of Animals in Ophthalmic and Vision Research, with approval of the Institutional Animal Care Committees at the University of Utah and Wright State University. Male rats were obtained at postnatal-day 21 (P21) and most were cyclic-reared in dim light (20–40 lux) on a 12–12 cycle in normal phase. At P60 they were dark adapted overnight and then exposed to intense visible light in green plexiglas chambers (490–580 nm light) beginning at 9 AM [11] in most cases. Some animals were used to assess the effects of phase onset. Similarly, a small set of animals were dark-reared to assess light history. Light exposures were for 24 or 48 h in most cases, to induce approximately 50 or 80% visual cell loss, respectively [10-12]. Following light exposure (pLX), all animals were returned to the dim cyclic light environment and maintained for 0, 14, 60, 120, or 240 days (i.e., pLX 0 to pLX 240). Animal tissues were rapidly harvested after gaseous carbon dioxide induced euthanasia.

**Table 1:** Light induced retinal damage animal cohorts.

**Sample**	**pLX days**					
	**0**	**14**	**60**	**120**	**240**	**Totals**
Controls	10*	6**	2	2	2	22
3 h pLX (DR)	9#	9#	0	0	0	18
8 h pLX (CR)	9#	9#	0	0	0	18
24 h pLX (CR)	3	0	4	3	3	13
48 h pLX (CR)	3	0	6	3	3	15
Totals	34	24	12	8	8	86

This table summarizes the experimental animal use in this study. The rows are the light treatments (see key) and the columns are the number of post-light exposure survival days (pLX). Each entry is the number of animals analyzed at each treatment vs survival combination. Abbreviations: pLX is post-light exposure, DR is dark-reared, CR is cyclic-reared, the asterisk designates 3 DR & 7 CR, the double asterisk designates 3 DR & 3 CR, and the sharp (hash mark) indicates 3 LX animals each initiated at 0100, 0900 or 1700 h.

### Sample processing

Eyes were immersion-fixed overnight to several weeks in buffered 2.5% glutaraldehyde/1% formaldehyde and resin embedded as previously described [27]. All samples were thin sectioned at 200–250 nm into serial arrays [29] and probed with IgGs (Table 2) targeting 4-aminobutyrate (GABA, γ), L-arginine (R), L-aspartate (D), L-glutamate (E), L-glutamine (Q), glutathione (J), glycine (G), and taurine (τ). All small molecule IgGs (except anti-R) were obtained from Signature Immunologics, Inc. (Salt Lake City, UT). Anti-R was developed by the Marc laboratory [28]. Rod opsin was probed with Rho 1D4 (a gift of Dr. Robert Molday of the University of British Columbia) and cone opsin probed with anti-LWS1 opsin (red/green opsin) from Millipore Corp. (product number AB-5405; Temecula, CA). The use and dilutions of most of these IgGs have been previously described [5] and our protocols are detailed at MarcLab. Anti-CRALBP was a gift from Dr. Jack Saari of the University of Washington and was used at 1:1000 from serum. All IgGs were visualized via silver-intensification of 1.4 nm gold granules conjugated to goat anti–rabbit or goat anti–mouse secondary IgGs (Table 2). Rat eyes are usually too large to section at 200 nm thickness for many serial sections, so most eyes were segmented into three arcs (ventral, central and dorsal) along the vertical axis through or near the optic nerve head. Sample processing was masked: neither the tissue processing, sectioning, immunochemical nor image capture staff knew the provenance or history of the samples. This resulted in over 400 blocs for sectioning from the 86 rats and over 6000 probed sections.

### Imaging and analysis

Images were captured as 8-bit, 1388-pixel 1036-line frames under voltage-regulated tungsten halogen flux with a Peltier-cooled camera (Fast 1394 Qicam; QImaging, Burnaby, BC, Canada) and autotiled with a montaging system (Syncroscan; Synoptics Inc., Frederick, MD) and an automated stage (Märzhäuser Wetzlar GmbH, Wetzlar, Germany) [5]. Images were acquired at nominal resolutions of 182 nm/pixel with a 40x oil immersion plan fluotar objective and 117 nm/pixel with a 63x oil planapochromatic objective. Final tiling and multimodal registration were performed with IR-tweak, a multi-platform registration application based on a Thin Plate Spline transform. More information about IR-tweak and other related microscopy image processing applications can be found at the Koshevoy Laboratory at University of  Utah.

Data sets were comprehensively scanned and nineteen remodeling attributes scored for every specimen (Table 3). The percentage of the animals in each group expressing the feature were tabulated. None of these remodeling features are ever observed in normal retinas and we have visualized data from over 100 normal mice, 50 rats, 200 rabbits, 12 primates, and 3 normal human retinas. Thus the occurrence of even a single instance of a glutamine-rich glial seal, for example is highly anomalous and highly significant. If we take the conservative example of 10 control rats never showing a glutamine-rich seal, then the estimate of the control incidence must be <0.1/animal. Then, finding glutamine-rich seals in 52 of 76 light exposed animals (which includes the day 0 animals) has a binomial probability of <3×10^−34^. Moreover, as we will show, each eye is its own control as patches rods and cones always survived in ventral regions of the eye and could be directly compared with damaged zones within eyes. In these instances we use quantitative imaging with curvilinear paths (ImageJ) to profile small molecule signals across transition zones between survivor and LIRD retina. All the remodeling events reported here are highly significant (p<0.001 in most cases) by a binomial calculation based on control incidence rats.

Individual cell classes were visualized as rgb mapped additive multispectral images [28]. These are termed ‘merged’ images in confocal imaging, though this is a mathematically incorrect term. Thus a mixture of taurine, glutamine and glutamate displayed as additive red, green, and blue channels is properly referred to as a τQE:rgb mapping [28]. Probability density histograms and spatial profiles of molecular signals in each cell class were extracted as previously described [27]. No image transforms were allowed in analysis: no filtering or scaling was used. An image editing system (PhotoShop CS3; Adobe Systems, Mountain View, CA) was used for final image display using max-min linear contrast stretches of each channel (typically with minimum and maximum pixel values of 30 and 220, respectively). All images were created with history logging embedded in the metadata. Some image processing analyses required developing new strategies to map structures. For example, relative hole frequency was estimated by the following procedure: (i) warp the IPL to a straight band; (ii) threshold the IPL to pixel value (PV) 168 isolate holes; (iii) cut the IPL into 3 μm strips spanning the width of the IPL; calculate the average PV of each strip. This yields the relative frequency of holes. Glutamine signals in Müller cell (MC) end feet were profiled in the warped images and those in the choroid profiled directly without warping using a single pixel width profile in ImageJ, filtered with a 2 μm boxcar average.

## Results

### Overview

**Figure 1:**
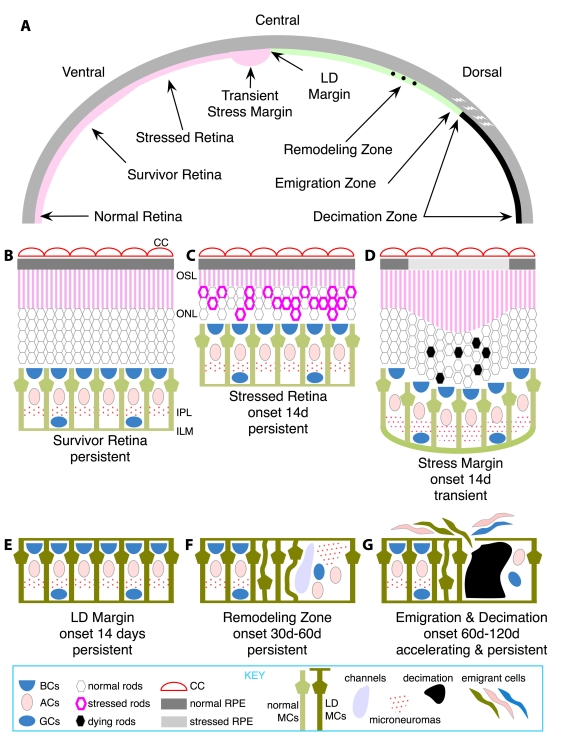
A summary of the key attributes of light-induced retinal damage across the albino rat retina. **A:** The dorsoventral gradient of alterations manifested over time. At late stages, normal retina is restricted to ventral retina near the ora terminalis. The bulk of intact retina is (**B**) survivor retina with shortened photoreceptors. At early stages, zones of (**C**) stressed retina with stressed photoreceptors are large but then contract to a thin rim near the light damage (LD) margin. Also at early stages, transient buckling (**D**) appears at the junction of intact retina and the LD margin (**E**) as photoreceptors continue to produce outer segment material, but retinal pigmented epithelium (RPE) cells are compromised. LD retina forms (**F**) remodeling zones with internal revisions such as neuronal migration, microneuromas and fluid channel formation; (**G**) emigration sites with aggressive migration of retina cells into the remnant choroid, leaving behind decimated zones with severely depleted retinas. Abbreviations: BCs represents bipolar cells, ACs represents amacrine cells, GCs represents ganglion cells, CC represents choriocapillaris, MCs represents Müller cells.

**Figure 2:**
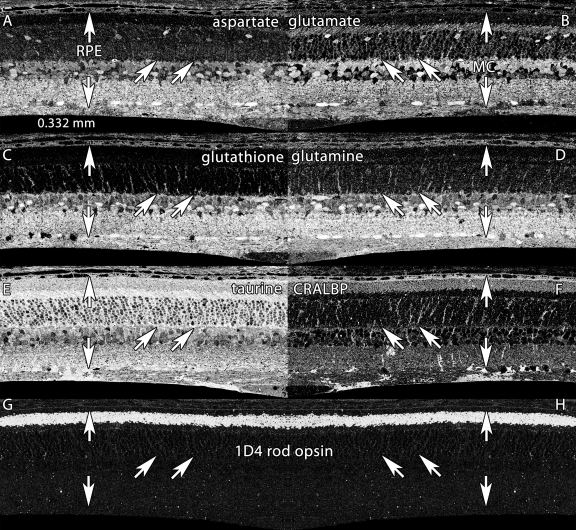
Basic molecular signatures in the normal albino rat retina. Visualization: Quantitative gray-scale images displayed as intensity in mirror-image pairs (AB, CD, EF, GH). Up arrows, retinal pigment epithelium (RPE); down arrows, Müller cell (MC) end feet; oblique arrows mark the border of the outer nuclear and outer plexiform layers. Panels are 0.332 mm wide. **A:** Aspartate is high in the RPE, in cone photoreceptors and various neurons of the neural retina but low in rods. **B:** Glutamate levels are high in neurons and lower in MC. **C:** Glutathione levels are high in the RPE and MCs, but very low in photoreceptors. **D:** Glutamine is high in all cells, but especially MCs. **E:** Taurine is high in the RPE, photoreceptors and MCs, and somewhat lower in neurons. **F:** cytosolic retinal binding protein (CRALBP) is expressed throughout MCs and the RPE, with significant levels in the outer segment layer. **G, H:** Mirror image pairs of rod opsin signals visualized with mAb rhodopsin 1D4. Note the extremely low somatic levels of rod opsin. Sample metadata: Sprague-Dawley (SD) Rat, age 60 d, animal #P60–1L-0, left eye, no LX, bloc code 6484, slide code 5251.

**Figure 3:**
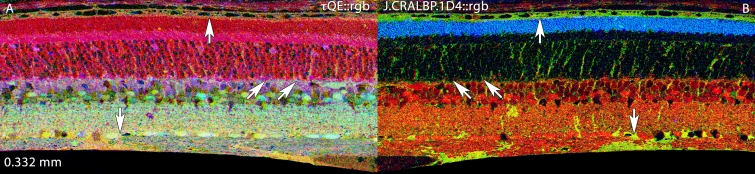
Basic molecular signature mixtures in the normal albino rat retina. Visualization: computational molecular phenotyping (CMP) images displayed as intensity in a mirror-image pair. Up arrows, RPE; down arrows, Müller cell (MC) end feet; oblique arrows mark the border of the outer nuclear and outer plexiform layers. Scale: All panels are 0.332 mm wide. **A:** Taurine (τ), glutamine (Q), glutamate (E) mixtures in a τQE::rgb mapping. This reveals the normal magenta (high τE) mix of photoreceptors and gold (high τQ) mix of MCs. **B:** Glutathione (J), cytosolic retinal binding protein (CRALBP) and rod opsin (1D4) in a J.CRALBP.1D4::rgb mapping. This segments the normal retina into spectrally distinct RPE, outer nuclear layer, neural, and MC compartments. Sample metadata: Sprague-Dawley (SD) Rat, age 60 day, animal #P60–1L-0, left eye, no LX, bloc code 6484, slide code 5251.

The prevalence of twenty molecular and structural indices of early stress, fundamental remodeling and late-stage retinal decimation are summarized in Table 3. The indices are chronologically ordered and give a broad view of the speed and complexity of remodeling. Figure 1 serves as a guide to the global architecture of LIRD remodeling over retinal space and survival time, placing the following detailed descriptions of each phase in context. Though stress signals are initially panretinal (see below), concrete LIRD defined by severe photoreceptor loss and Müller cell remodeling begins focally, expands and remains a regional process. The kinetics of that expansion is not the focus of our work, but it is clear that discrete LIRD foci ranging from <0.1 mm to over 1 mm in size emerge by pLX 14 and can emerge sooner. Where survivor retina and LIRD remodeling zones abut, the retina is sometimes thrown up into buckles or manifests increases in outer segment length. As we will discuss below, our evidence indicates early metabolic stress of RPE cells in these regions. However, these regions are transient and absent from late-stage LIRD eyes. Similarly, LIRD eyes immediately manifest photoreceptor stress markers such as severe opsin delocalization immediately after light exposure, but this too largely disappears. Survivor retina possesses fewer and much shorter rods than normal retina but appears metabolically healthy and stable in all other aspects of neural structure and metabolic signatures, and closely mimics the molecular profiles of normal retina (Figure 2 and Figure 3). At advanced stages, the margins between LIRD and survivor retina are precise and show no gradations. Retina is either normal or remodeled and the decisive factor is the presence of photoreceptors, regardless of how few or how small. Complex remodeling zones are abundant in LIRD foci by pLX 60. These zones are completely and unambiguously defined by complete conversion of all Müller cells to an anomalous high glutamine state characteristic of many retinal degeneration phenotypes [1]. Further, remodeled Müller cells are severely depressed in taurine content, suggesting that their abilities to osmoregulate are compromised. This is consistent with the emergence of large numbers of fluid channels precisely within LIRD foci and never in survivor retina. Focal instances of new neurite formation known as microneuromas [1,6] and, less commonly, neuronal migration within the retina reach their peak at pLD 60–120 and then decline. The most dramatic and devastating effect of remodeling is emigration of Müller cells and neurons from the retina into the survivor choroid, leading to extensive decimation of the retina. In these zones, retinal metabolic signatures are severely depressed and Q signals (and others) drop precipitously. These summary data argue that LIRD leads to death of the neural retina; but only in LIRD zones, not in survivor retina.

### Early stress

**Figure 4:**
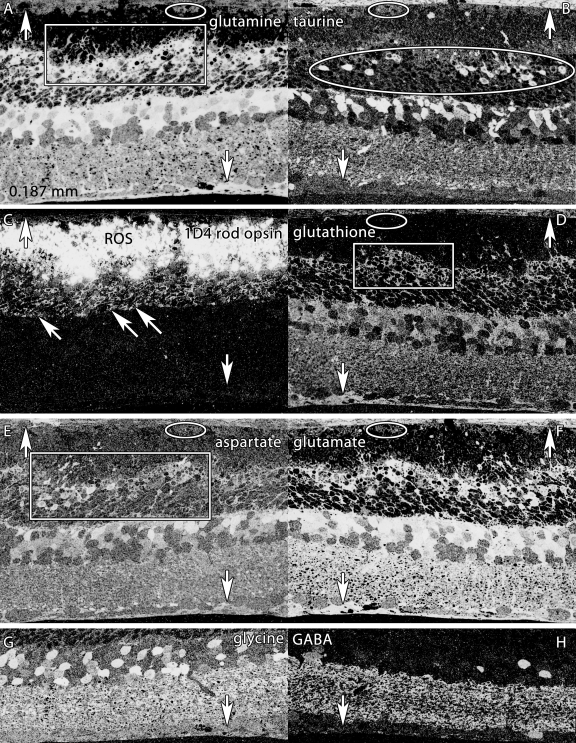
Altered molecular signatures immediately after 48 h of light exposure. Visualization: Quantitative gray-scale images displayed as intensity in mirror-image pairs (**AB, CD, EF, GH**). Up arrows, choroid-retinal pigment epithelium (RPE) interface; down arrows, Müller cell (MC) end feet; oblique arrows mark the border of the outer nuclear and outer plexiform layers. Scale: All panels are 0.187 mm wide. **A:** Glutamine signatures are elevated in MCs, with hypertrophy of distal MC processes at the external limiting membrane (box). The RPE layer is severely damaged, with only a few distinct cells (oval). **B:** Massive taurine depletion in MCs (down arrows) and abnormal elevation in photoreceptors (oval) and bipolar cells. **C:** 1D4 rod opsin reveals extreme disorganization of rod outer segments and extensive mislocalization of rod opsin into rod somas. **D:** Glutathione signatures highlight the disorganization of MC processes in the outer retina. **E:** Aspartate signals are abnormally high in rod inner segments. **F:** Glutamate signals in particular are abnormally elevated in MCs. **G, H:** Glycine and γ-aminobutyric acid (GABA) signals seem essentially normal, with no evidence of ischemia or excitotoxicity. Sample metadata: SD Rat, age at LX 60 d, animal #P60–1L-48, left eye, 48 h LX, harvested at 0 days pLX, bloc code 6464, slide code 3548b.

**Figure 5:**
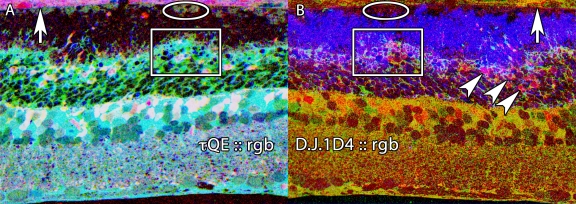
Altered molecular signature mixtures immediately after 48 h of light exposure. Visualization: CMP images displayed as intensity in a mirror-image pair. Up arrows, choroid-RPE interface; oblique arrowhead mark stressed rods. Abbreviations: IPL represents inner plexiform layer, INL represents inner nuclear layer. Scale: All panels are 0.187 mm wide. **A:** τQE::rgb mapping demonstrates the remarkable inversion of the MC profile from normal high τQ, low E to abnormal low τ, high QE and severe depletion of the RPE layer (ovals). **B:** D, J**:** 1d4::rgb mapping concurrently displays rod opsin mislocalization, high aspartate stress signals in selected rods (oblique arrowheads), and severe disruption of the external limiting membrane-photoreceptor interface (boxes). Sample metadata: SD Rat, age at LX 60 d, animal #P60–1L-48, left eye, 48 h LX, harvested at 0 days pLX, bloc code 6464, slide code 3548b.

SD rats manifest photoreceptor, Müller cell and RPE stress immediately after light exposure. Stress signals are metabolomic and proteomic (Figure 4 and Figure 5). Rod outer segments are severely disrupted and elevated glutamine, glutathione, and glutamate signals are found in the swollen distal processes of Müller cells at the external limiting membrane. At the same time, taurine levels are severely depressed. The structural damage to photoreceptors is accompanied by mislocalization of rod opsin to inner segments (compare Figure 2G and Figure 3B with Figure 4C and Figure 5B). We have identified several new markers of photoreceptor metabolic stress but here address only the anomalous elevation of L-aspartate in rods of pLX 0 and 14 animals (Figure 4 and Figure 5). As far as we can discern, aspartate “stress” is nearly panretinal at pLX 0 but resolves to a ring of scattered remnant stressed photoreceptors at the LIRD margin by day 60, and slowly disappears. The mislocalization of opsins follows the same pattern: first panretinal, then circumferential to the LIRD focus, and then largely, but not completely absent.

**Figure 6:**
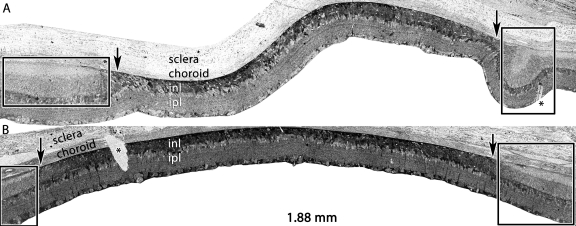
Emergence of the high glutamine MC seal by pLX 14. Visualization: Quantitative gray-scale images of glutamine signals displayed as density in two separate retinas, one displaying buckled margins (**A**) and the other with smooth margins (**B**). Down arrows, choroid-retina interface bracketing the LD zone; asters, section damage. Scale: The panels are 1.88 mm wide. Sample A metadata: SD Rat, age at LX 60 d, animal #VO 01–56 SDCRLD8–5PM-2WK-2L, left eye, 8h LX onset at 5PM, harvested 14d pLX,bloc code 6547, slide code 3304. Sample B metadata: SD Rat, age at LX 60 d, animal #VO 01–69 SDDRLD3–5PM-2WK-3L, left eye, 8h LX onset at 5PM, harvested at 14 days pLX, bloc code 6560, slide code 3319.

Glial stress signals are equally rapid, with Müller cell glutamate levels reaching higher than ever observed in any other condition. Glutamine and glutathione signals are extremely elevated in parallel (as well as several other signals). However, this is not a non-specific trapping of small molecules, as taurine levels drop dramatically, producing cyan τQE signatures (Figure 5A) vastly different from the yellow-orange tQE patterns of normal Müller cells (Figure 3A) in most vertebrates [28-31]. The elevation of glutamine contents is a persistent feature of the LD zone, while survivor retina reverts to the a nearly normal level (Figure 6). The margins of LIRD foci sometimes manifest retinal buckling at pLX 14 (Figure 6A) while others do not (Figure 6B). This is not a tissue processing defect as it never occurs in normal tissue and disappears in the late-stage LIRD. Neither the nearby retina nor RPE is deformed, but the RPE is clearly metabolically stressed as the normal glutamine signals of healthy RPE radically drop in these marginal zones even in the presence of photoreceptors.

### The loss of the photoreceptor-retinal pigment epithelium-choriocapillaris complex

**Figure 7:**
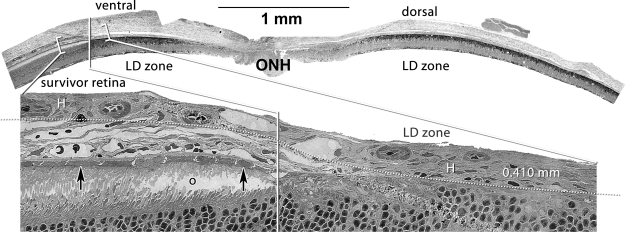
Persistent remodeling of the choroid-retinal pigment epithelium (RPE) interface over a dorsoventral gradient. Visualization Top: Quantitative gray-scale images of glutamine signals displayed as density. Visualization Bottom: Serial section stained with toluidine blue. Up arrows, RPE; o, disrupted rod outer segments in the corner of the eye-shaped survivor zone photoreceptor outer segment layer; H, Haller’s layer of the choroid. Scale: The top panel is over 5 mm long and the bottom image is 0.410 mm wide. The enlarged view shows the complete loss of the choriocapillaris and RPE in the LD zone and complete retention (arrows) in the survivor zone. The large vessel Haller’s layer of the choroid directly abuts the remnant retina in the (LD zone dotted line). ONH, optic nerve head. Sample metadata: SD Rat, age at LX 60 d, animal #VO 01–72 SDDRLD3–1AM-2WK-3L, left eye, 3 h LX starting at 1AM, harvested at 14 days pLX, bloc code 6563, slide code 3322.

LIRD kills three classes of cells concurrently: photoreceptors, the RPE and the endothelia of choriocapillaris (Figure 7). While we have no data on transient apoptosis rates in the rat, all three cell classes manifest Terminal deoxynucleotidyl transferase-mediated dUTP nick end labeling (TUNEL) signals in albino mice within 24 h post-LIRD (unpublished data). This border of RPE-choroicapillaris ablation is extremely precise in pLX 14 animals and beyond (Figure 6 and Figure 7). The Müller cells form the distal margin of the retina in LIRD foci. There is an extremely sharp transition between healthy RPE and no RPE; between healthy endothelia and no endothelia; between normal τQ signatures in Müller cells of survivor retina and abnormal low taurine - high glutamine signatures in Müller cells of the LIRD zone.

### Classical remodeling

**Figure 8:**
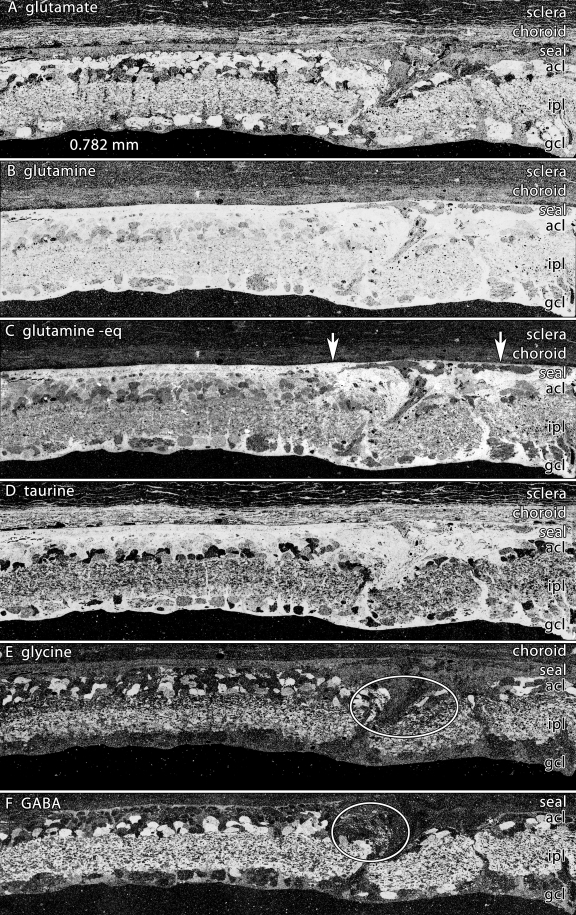
Neuronal migration columns and microneuromas in light induced retinal damage. Visualization: Quantitative gray-scale images of molecular signals displayed as intensity. Down arrows mark the remnant retina-choroid interface, arrows delimit an intense remodeling zone with disruptions in the integrity of the MC glutamine-rich seal and emergence of both migrating neurons and clusters of new neurites (ovals). Abbreviations, acl is amacrine cell layer, ipl is inner plexiform layer, gcl is ganglion cell layer. Each panel is 0.782 mm wide. **A:** Glutamate signals reveal a distinctive non-neural seal between glutamate-rich remnant bipolar cells and the choroid and a mixture of high-glutamate elements in the remodeling column. **B:** Glutamine signals are extremely high in the remnant retina and lower in the choroid. The signals in the seal and migration column are exceptionally high, with some darker islands within the column. **C:** Glutamine histogram equalization (eq) highlights the seal, the column and the lower glutamine levels of cells between the arrowheads. **D:** Taurine levels demonstrate recovery from the early depletion at 14–50 pLX. **E:** Glycine signals highlight migrating neurons within the column and new processes streaming distally (oval). **F:** γ-aminobutyric acid signals display the development of new amacrine cell neurites in the column. Sample metadata: SD Rat, age at LX 60 d, animal #P180–2L-48–120, right eye, 48 h LX, harvested at 120 days pLX, bloc code 6628, slide code 3535.

The LIRD model recapitulates the major features of remodeling in inherited degenerations by manifesting glial hypertrophy and reorganization of neural retina (Figure 8). By pLX 120, many neurons have generated new processes that course erratically through the remnant inner nuclear layer and occasionally form visible aggregates we have previously described as microneuromas (γ-aminobutyric acid (GABA) and glycine panels of Figure 8). Similarly, clusters of anomalously positioned amacrine and ganglion cells near hypertrophic Müller cells and invasive vasculature show pathologic migration patterns.

**Table 2:** Immunoreagents

**Primary IgG**	**Dilution**	**Code/SKU**	**Host**	**Source**
Anti-L-arginine	1:1000	R	Rabbit	Marc Laboratory, University of Utah
Anti-L-aspartate	1:2000	D100	Rabbit	Signature Immunologics Inc., Salt Lake City, UT
Anti-L-glutamate	1:32000	E100	Rabbit	Signature Immunologics Inc., Salt Lake City, UT
Anti-glycine	1:4000	G100	Rabbit	Signature Immunologics Inc., Salt Lake City, UT
Anti-glutathione	1:4000	J100	Rabbit	Signature Immunologics Inc., Salt Lake City, UT
Anti-glutamine	1:4000	Q100	Rabbit	Signature Immunologics Inc., Salt Lake City, UT
Anti-taurine	1:16000	TT100	Rabbit	Signature Immunologics Inc., Salt Lake City, UT
Anti-GABA	1:32000	YY100	Rabbit	Signature Immunologics Inc., Salt Lake City, UT
Anti-CRALBP	1:1000	CRA	Rabbit	Gift of J Saari, University of Washington
Anti-rhodopsin	1:8000	Rho 1D4	Mouse	Gift of R Molday, University of British Columbia
Anti-LWS opsin	1:3200	AB5405	Rabbit	Millipore Inc., Billerica MA
Secondary IgG	Dilution	Conjugate	SKU	Source
Goat anti-Rabbit	1:100	1.4 nm gold	2003	Nanoprobes Inc., Yaphank, NY
Goat anti-Mouse	1:100	1.4 nm gold	2001	Nanoprobes Inc., Yaphank, NY

This table summarizes the antibodies used in this study. The rows are the individual IgGs and the columns denote the dilution, identifier code, host and source for each.

### Extreme remodeling

**Figure 9:**
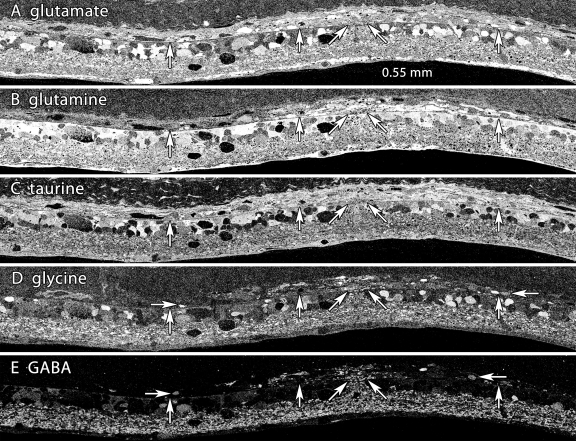
Eruption of neurites into the choroid. Visualization: Quantitative gray-scale images of molecular signals displayed as intensity. Up arrows, remnant retina-choroid interface; horizontal arrows, neurons and neurites migrating in the choroid; angled arrows, eruption site. Each panel is 0.550 mm wide. **A:** Glutamate signals do not readily reveal any interruption in the MC seal. **B:** Glutamine signals generally indicate that the MC seal is confluent, though subtle variations in signal strength between the slanted arrows suggests an altered environment. **C:** Taurine signals do not readily reveal any interruption in the MC seal. **D:** Glycine signals indicate an eruption. Many glycinergic structures are located in the choroid distal to the arrows. **E:** γ-aminobutyric acid signals signals clearly indicate amacrine cell processes are entering the choroid. Sample metadata: SD Rat, age at LX 60 d, animal #P240–3L-48–240, left eye, 48 h LX, harvested at 240 days pLX, bloc code 6693, slide code 3528.

**Figure 10:**
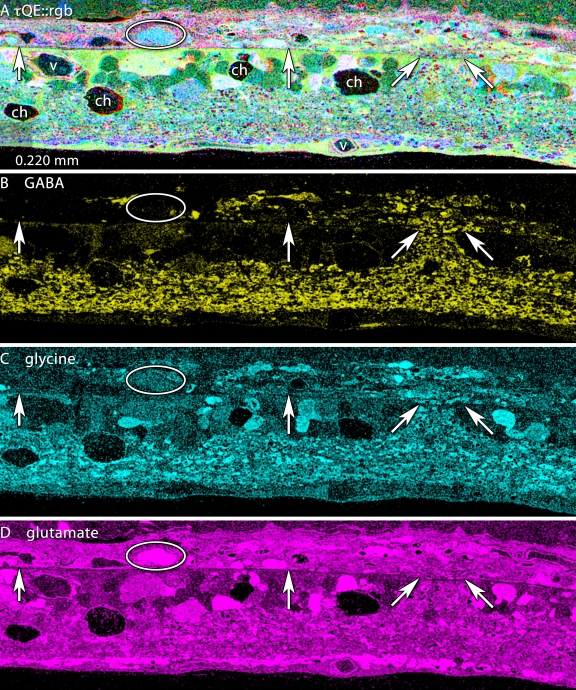
High resolution mapping of the eruption of neurites into the choroid. Visualization: CMP and cyan-magenta-yellow (cmy) mapped images displayed as intensity channels. See Marc and Cameron [29] for an explanation of cmy-mapping. Up arrows, remnant retina-choroid interface; angled arrows, eruption site; ovals, migrating glutamate+ neurons; ch, fluid channels; v, capillary. Each panel is 0.220 mm wide. **A:** τQE::rgb mapping clearly delineates the border between the remnant retina (yellow-green) and the choroid (pink). Distinctive QE+ cyan somas characteristic of ganglion cells are in the choroid (oval). **B:** γ-aminobutyric acid (GABA):yellow mapping clearly reveals both neurites and fusiform GABA+ somas deep in the choroid. **C:** Glycine::cyan mapping similarly shows glycine+ profiles in the choroid. **D:** Glutamate::magenta mapping shows that, while it is not possible to selectively visualize glutamate+ profiles exiting the retina, large glutamate+ profiles are now abundant in the choroid. Sample metadata: SD Rat, age at LX 60 d, animal #P240–3L-48–240, left eye, 48 h LX, harvested at 240 days pLX, bloc code 6693, slide code 3528.

**Figure 11:**
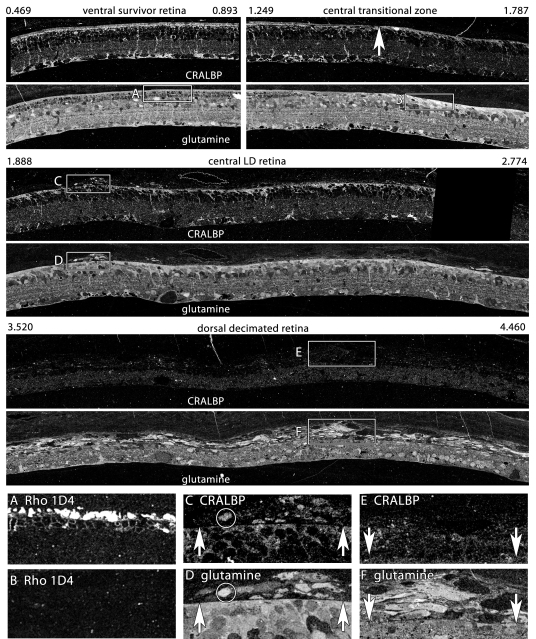
Glutamine and cytosolic retinal binding protein (CRALBP) gradients in survivor, light damage, and decimated retina. Visualization: Quantitative gray-scale images of molecular signals displayed as intensity. Boxes indicate selected regions illustrating characteristic local features. The data set derives from a single 200 nm section over 5 mm long; segments between 0.496 and 4.46 mm are shown. 0.496–0.893 mm: Ventral survivor retina displays normal cytosolic retinal binding protein (CRALBP) and glutamine signatures. Rod opsin signals of boxed region are shown in panel **A:** 1.249–1.787 mm: Central survivor-light damage (LD) transition (upward arrow) shows Müller cell (MC) hypertrophy and elevated distal seal CRALBP and glutamine signals in LD zone. Rod opsin signals of boxed region are shown in panel **B:** Upward arrow denotes seal border. 1.888–2.774 mm: Dorsal LD retina has a nearly confluent seal with focal emigration clusters. CRALBP and glutamine signals of early emigrant cells are compared in panels **C, D:** 3.520–4.460 mm: Dorsal decimated retina shows loss of the glutamine seal and massive emigration. Over 50% of the retinal mass has moved into the choroid. CRALBP and glutamine signals of late emigrant cells are compared in panels **E** and **F**: **A:** Rod opsin rhodopsin 1D4 in survivor retina B: Rod opsin rhodopsin 1D4 in LD retina **C**: CRALBP in early emigrant cells of LD zone. Upward arrows denote seal. **D:** Glutamine in early emigrant cells of LD zone. Upward arrows denote seal. **E:** CRALBP signals are depressed in late emigrant cells of LD zone. Downward arrows denote seal. **F:** Glutamine signals remain high in late emigrant cells of LD zone. Downward arrows denote seal. Sample metadata: SD Rat, age at LX 60 d, animal #P180–2R-48–120, right eye, 48 h LX, harvested at 120 days pLX, bloc code 6627, slide code 5223.

Fluid channel evolution is restricted to the LIRD zone, but can be very severe, with survivor retina adjacent to the LIRD zone appearing completely normal (Figure 9). We have documented such channels in the transgenic P23H rat retina, in the RCS rat and many human RP retinas (unpublished data). The key feature that differentiates the LIRD model from all others is the eruption of processes from retinal neurons and ultimate emigration of Müller cells and neurons of all classes from the retina into the choroid (Figure 9 and Figure 10). Figure 9 illustrates early focal ruptures in the Müller cell-choroid interface and emigration of neurons from the retina. Elements with Müller cell-like high glutamine levels as well as neuronal processes (Figure 9) and neuronal groups of all signature classes can be found in the choroid. Most of these emigrant cells are fusiform and have clearly lost their characteristic shapes, but they retain their essential neurochemical profiles. Initially, small clusters of cells can be found in the choroid opposite remodeled Müller cells with extremely robust glutamine signals and a dense distal seal even at pLX 60 and abundantly at pLX 120 (Figure 11), implying that exit from the retina is actually an early process and that cells may translocate long distances. CRALBP is a distinctive marker for Müller cells and colocalization of CRALBP with glutamine reveals discrete patterns of Müller cell transformation. In survivor zones where rods persist (albeit shortened), Müller cell CRALBP-glutamine signatures are normal (Figure 11A). In the LD zone lacking photoreceptors, both CRALBP and glutamine levels are significantly elevated at the distal seal (Figure 11B). Figures 11C,D show that small clusters of emigrant cells with Müller cell CRALBP-glutamine signatures have formed above LD zones. However, in more dorsal retina (presumably the most severely damaged) emigration is so severe that over half the mass of the glutamine signal in the retina has moved to the choroid leaving a severely decimated retina. However, emigrant cells have severely down-regulated CRALBP expression (Figure 11E) despite preservation of the glutamine signal (Figure 11F).

**Figure 12:**
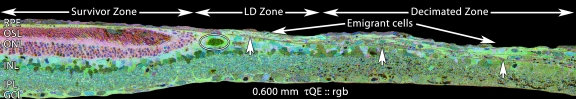
Detailed Survivor-light damage (LD)-Decimation gradient. Visualization: CMP images displayed as intensity channels. Up arrows, remnant retina-choroid interface; oval, fluid channels. The panel is 0.600 mm wide. τQE::rgb mapping shows the roughly normal spectral signatures of the retinal pigment epithelium (RPE), photoreceptors and neural retina, with a very small LD zone and a massive decimated zone with weak retinal glutamine signals and large numbers of emigrant cells with high QE signatures. Sample metadata: SD Rat, age at LX 60 d, animal #P240–2L-24, left eye, 24 h LX, harvested at 240 days pLX, bloc code 6685, slide code 3518.

**Figure 13:**
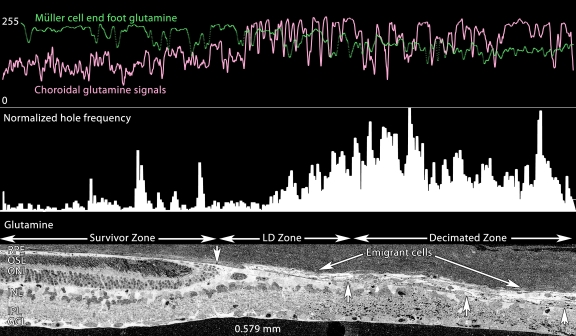
Glutamine profiles across the Survivor-light damage (LD)-Decimation gradient. Visualization: Quantitative gray-scale images of glutamine signals displayed as intensity. Up arrows, remnant retina-choroid interface; downward arrow is the small high glutamine seal region. The bottom panel is 0.579 mm wide and shows isolated glutamine gradients in retina, retinal pigment epithelium (RPE), choroid, seal, emigrant and decimated zones. All decimated zones show large numbers of holes (see Methods). Sample metadata: SD Rat, age at LX 60 d, animal #P240–2L-24, left eye, 24 h LX, harvested at 240 days pLX, bloc code 6685, slide code 3518.

**Figure 14:**
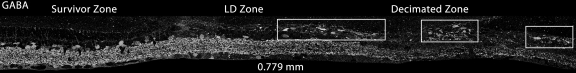
γ-aminobutyric acid (GABA) profiles across the survivor-light damage (LD)-Decimation gradient. Visualization: Quantitative gray-scale images of GABA signals displayed as intensity. Boxes denotes large aggregates of emigrant GABA+ cells and processes in the decimated zone. The panel is 0.779 mm wide. Note the severe loss of GABA signals in the decimation zone. Sample metadata: SD Rat, age at LX 60 d, animal #P240–2L-24, left eye, 24 h LX, harvested at 240 days pLX, bloc code 6685, slide code 3518.

In time, pure LD zones with strong glutamine-rich Müller cell seals become small, so that at pLX 240, decimated retina dominates. Figure 12 displays a precise serial transition from survivor retina with a largely normal τQE signature, to a narrow LD zone, and then a large region of decimated retina with extremely weak τQE signals altogether. This is not an artifact of processing as cells in the choroid have exceptionally strong signals. The dissipation of retinal glutamine signals is quantitatively demonstrated in Figure 13, where the glutamine contents of the Müller cell end feet and choroid are profiled in parallel, demonstrating reversed gradients. This suggests that many of the glutamine rich cells of the choroid (which never appear in normal choroid) are migrant Müller cells. Indeed, the subjacent retina is replete with a large number of holes. Quantitatively, decimated retina has an integrated frequency of such holes sixfold greater than survivor retina 0.15 mm away. This decimation is neural as well. Figure 14 demonstrates the depletion of GABA signals in the decimated zone of the retina with simultaneous emergence of a large number of GABA+ profiles in the choroid.

**Figure 15:**
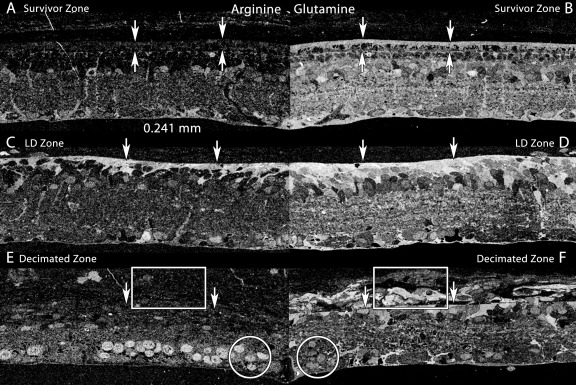
Arginine and glutamine profiles across the Survivor-light damage (LD)-decimation gradient. Visualization: Quantitative gray-scale mirrored images of molecular signals displayed as intensity. Boxes denotes aggregates of emigrant glutamine+ cells and processes in the decimated zone. Downward arrows mark the choroid-retinal pigment epithelium (RPE, AB) or choroid-retina interface (CDEF). Upward arrows marks the external limiting membrane. Circles mark high-arginine ganglion cells. Each panel is 0.241 mm wide. **A:** Arginine levels in survivor retina are modest and mostly in Müller cells and some amacrine cells, similar to normal retina. **B:** Glutamine levels levels in survivor retina are high in Müller cells, the RPE and many other cell types, similar to normal retina. **C:** Arginine levels in the LD zone are elevated in Müller cells associated with the distal seal. **D:** Glutamine levels in the LD zone are elevated in Müller cells associated with the distal seal. **E:** Arginine levels in the decimated zone and emigrant cells collapse in Müller cells (box) but are elevated in the nuclei of remnant ganglion cells (circle). **F:** Glutamine levels in the decimated zone drop but remain high in emigrant Müller cells (box). Levels are modest in the nuclei of remnant ganglion cells (circle). Sample metadata: SD Rat, age at LX 60 d, animal #P180–2R-48, right eye, 48 h LX, harvested at 120 days pLX, bloc code 6627, slide code 5228.

The transformation of Müller cells is complex and involves more than the basic τQE signature. An additional, largely Müller cell-specific signal is arginine. Under normal circumstances in the mammalian retina, including survivor retina, Müller cell arginine levels are modest (Figure 15A) and slightly less than found in a unique set of amacrine cells. However, formation of the glial seal triggers a major increase in arginine levels, especially in processes near the seal (Figure 15C). This increase collapses in decimated retina and even though emigrant cells persist in maintaining high glutamine levels, arginine synthesis is halted (Figure 15E).

### Human geographic atrophy

**Figure 16:**
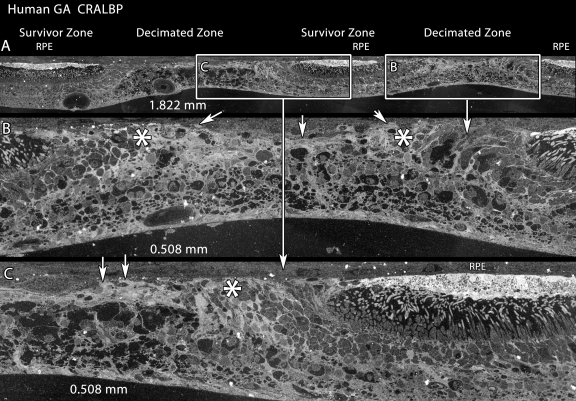
Human geographic atrophy mimics the light induced retinal damage phenotype. Visualization: Quantitative gray-scale images of cytosolic retinal binding protein (CRALBP) signals displayed as intensity. Boxes denote decimated zones; arrowheads indicate the uneven choroid-retina interface. Asters indicate the sharp survivor-decimated retina border. **A:** CRALBP signals displays three patches of retinal pigment epithelium (RPE) over survivor zones, with decimated retina between. Panel width 1.822 mm. **B, C:** CRALBP signals are elevated in presumed remnant Müller cells of the seal region (aster), but the zone between is severely depleted of cells and the choroid-retina interface is uneven. Panel widths 0.508 mm.Sample metadata: Human age related macular degenerations (AMD, geographic atrophy); FFB# 9331; age at harvest 81 y, post-mortem delay 9 h, fixation 1% formaldehyde - 2% glutaraldehyde - 0.1 M PB, 3 mm perimacular trephine, bloc code 8771, slide code 5350.

Collaborative studies on remodeling in AMD (with J.E. Hollyfield and M. E. Rayborn of the Cole Eye Institute, Cleveland Clinic, Cleveland, OH) have allowed to us visualize a remarkable parallel between atrophic zones in human geographic atrophy (GA) and decimated retina in LIRD. Figure 16 displays the CRALBP signal in 9 h post-mortem human GA retina. While small molecule signals have been lost due to this extended post-mortem delay, rod and cone opsins (not shown) as well as CRALBP retain excellent signal strength, revealing that the morphology and proteomic signatures of Müller cells in the gradient between human survivor and decimated retina is indistinguishable from late phase LIRD retina of the SD rat (e.g., Figure 12 and Figure 13).

## Discussion

The rodent LIRD model displays remodeling features common to a range of RP-like disorders, including formation of a distal Müller cell seal, fluid channels, migration of retinal neurons, and formation of microneuromas. These features emerge rapidly in LIRD (within pLX 60), ranking it among the fastest genetic models [1]. This is largely due to the fact that phase 1 and 2 remodeling in LIRD is regionally complete in weeks rather than months to years in RP-like disorders. However, phase 3 remodeling appears to progress with similar kinetics in all models upon loss of the cone photoreceptors [1]. LIRD validates the idea that remodeling can be initiated at any time and its emergence is independent of specific genetic or environmental triggers. LIRD also avoids complexities associated with the overlap of retinal development and degeneration onset in genetic models. However, LIRD is more complex in triggering extreme remodeling via cellular emigration. It is impossible to gauge the degree of neuronal death in LIRD-triggered remodeling as a vast cohort of cells literally leaves the retina. Upon breakdown in the choroid-retina barrier, cell emigration decimates the remaining retina, up to a point. The gradient between apparently functional survivor retina and decimated retina is extremely steep (within 0.1 mm) and closely resembles the pattern of survivor and decimated retina in atrophic AMD. This obviously raises the question of whether similar emigration events are involved.

### A new stress signal: aspartate

CMP broadly interrogates cellular functions such as energetics (glutamate, aspartate), heterocellular group transfer functions (glutamine), osmoregulation (taurine), redox regulation (glutathione), neural signaling states (glutamate, GABA, glycine), and polyamine biosynthesis (arginine). When augmented by probes targeting additional cell-specific probes (opsins and CRALBP), CMP signatures precisely report transformations or persistence across cell populations in a spatially quantitative manner that protein chemistry cannot begin to approach. During light-induced stress before remodeling, several changes can be observed. First, massive rod opsin mislocalization was observed immediately after both 24 h and 48 h light exposures. Rod opsin redistribution to the inner segment has been well documented [32-35] in a variety of retinal disease models. We also observed novel altered rod signatures and the one of most immediate interest is aspartate. While we have not established a mechanism for the aspartate increase in stress, we have previously observed aspartate elevations in rods after prolonged ischemia and during phase 2 of many inherited retina degenerations (unpublished data). The cellular switch for setting predominantly oxidative versus glycolytic energy modes (i.e., neuronal versus glial) is expression of the mitochondrial aspartate-glutamate carrier (AGC, SLC25A12) [36]. AGC is highly expressed in photoreceptors and, in its absence, the critical aspartate-malate shuttle is disabled [37]. An important biophysical attribute of AGC is its conversion to a channel-like uniporter (a mitochondrial exporter) upon mercurial modification of cysteine residues [38]. Mercury acts as a bis-coupling agent to produce R-S-Hg-S-R complexes, mimicking oxidation-induced R-S-S-R dithiol links [39]. Photoreceptor-specific proteins are known to display function-altering oxidative damage in LIRD and the thiol-specific reagent dimethylthiourea is a known protectant [40]. If AGC experiences oxidative dithiol formation, it will become a fast exporter of aspartate from mitochondrial stores. Further, parallel collapse of the mitochondrial α-ketoglutarate/malate transporter function will prevent cytosolic conversion of aspartate to glutamate, resulting in aspartate accumulation. While this is a circumstantial argument, it is testable and argues that aspartate signals could represent a precise pre-apoptotic stress marker.

### Remodeling in light-induced retinal damage

**Figure 17:**
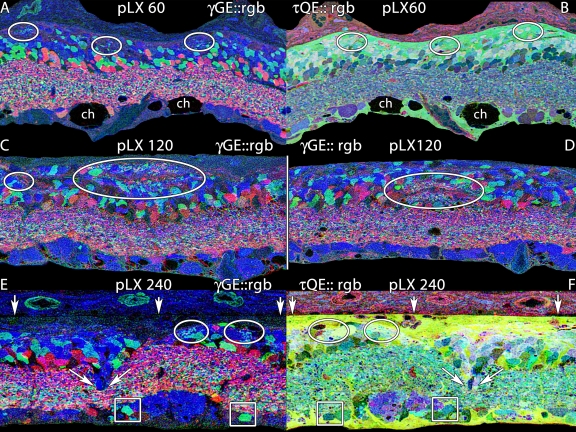
Microneuromas in light induced retinal damage at pLX 60, 120 and 240. Visualization: computational molecular phenotyping (CMP) images displayed as mirrored (AB, EF) or individual (CD) intensity channels. Down arrows, remnant retina-choroid interface; angled arrows, cell migration; ch, fluid channels; ovals, microneuromas. **A:** γGE::rgb mapping at pLX 60 reveals small microneuromas formed in part by amacrine cell neurites in the distal retina. Each panel is 0.256 mm wide.**B:** τQE::rgb mapping shows that the microneuromas are embedded in the distal seal region far from the inner plexiform layer.**C, D:** γGE::rgb mapping at pLX 120 displays larger microneuromas above (**C**) and within (**D**) the inner nuclear layer. Each panel is 0.256 mm wide.E. γGE::rgb mapping at pLX 240 reveals small microneuromas flanking a column of migrating neurons. Boxes highlight two misplaced glycinergic amacrine cells. Each panel is 0.170 mm wide.**F**. τQE::rgb mapping at pLX 240 reveals the complex cell types forming the migration column and that microneuromas are again close to the distal seal.

Our primary goal was to assess the chronology and scope of neural remodeling in LIRD as a non-genetic way to decouple remodeling from retinal development [9]. Importantly, the LIRD model recapitulates all the essential attributes of remodeling observed in human RP and animal models of retinal degenerations [1,7,21,41]. Figure 17 summarizes the progressive emergence of key remodeling features, beginning with the development of small microneuromas within the Müller cell seal by pLX 60 (Figure 17A,B), and continued expansion of microneuromas by pLX 120 both distal to (Figure 17C) and within (Figure 17D) the remnant inner nuclear layer. Finally, as in genetic models, frank movement of retinal neurons in migration columns develops, with the anomalous placement of glycinergic amacrine cells and bipolar cell clusters in the remnant ganglion cell layer (Figure 17D,E). This argues that the reorganization observed in human RP and animal models of inherited retinal degenerations is generic to photoreceptor deafferentation and not related to any specific gene defect. This is important for AMD, as we will note below. While most of the analyses of remodeling focused on 24–48 h LX models, we note that animals exposed for only 3 h (Figure 7) in the dark-cycle of their photoperiod (Table 1) also progress to phase 3 remodeling.

### Müller cell transformations

**Figure 18:**
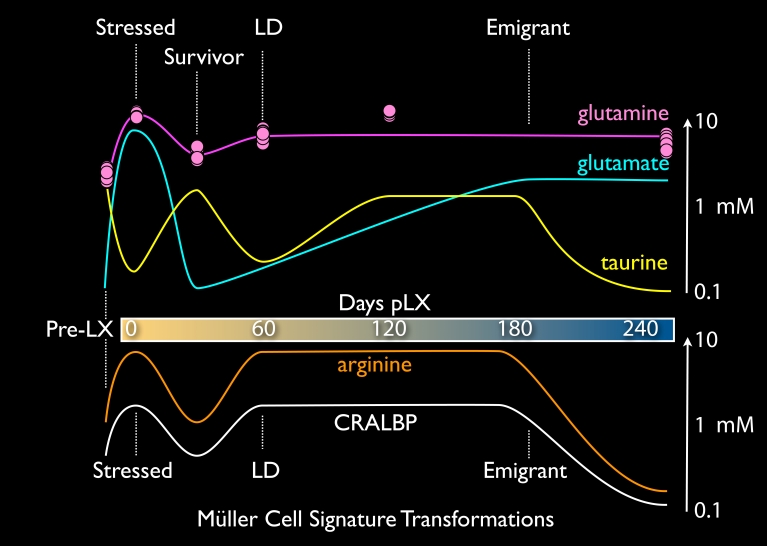
Complex Müller cell phenotypes in light induced retinal damage. The traces represent aggregate chronologic changes in mean concentrations of taurine, glutamine, and glutamate (the τQE signature at top) and arginine and cytosolic retinal binding protein (CRALBP) bottom. See text.

Müller cells are remarkable in their ability to display extremely diverse metabolic profiles. LIRD provides an exceptional platform to selectively drive them to different states: normal → stressed → transient survivor → remodeled → emigrant. Each state manifests a unique signature and importantly, our collection of LIRD retinas with complete survivor-LD-decimated gradients demonstrates that these variations cannot be attributed to inter-animal or technical variation. CMP was designed to clarify analysis by (1) standardizing probes and imaging platforms and (2) multiplexing retinas into single samples for concurrent analysis [28,29,42]. First, it is important to note that CMP can be precisely calibrated to report molecular concentrations in the 0.1–10 mM range [27,42]. Second, signals for different molecules are obtained from the same cell sources by virtue of CMP on serial 200 nm section arrays with precise image registration [29]. Thus, spatial gradients, stress and control samples can be summarized in a molecular chronology of Müller cell transformations tracked by τQE signatures, arginine and CRALBP signals (Figure 18). Each trace was determined from direct measurements and summarized as a complex spline (e.g., [43]). For example, the glutamine trace is accompanied by data point clusters from ten Müller cells at each condition. Similar very tight data point clusters define each trace, but are not shown for simplicity. As noted previously [27], metabolic profiles within a cell class are extremely precise and display little variability.

The Müller cell τQE signature changes in very discrete ways. Immediate LIRD stress evokes glutamate levels higher than any process other than direct in vitro loading of Müller cells with millimolar extracellular glutamate (unpublished data). Massive photoreceptor cell stress and death may generate a large glutamate efflux and Müller cell accumulation via the potent EAAT1 (SLC1A3) glutamate transporter [44]. A concurrent rise in glutamine content suggests that glutamine synthetase (GS) activity in Müller cells remains. Importantly, GS activity is not impaired by oxidative damage early in LIRD [40]. But remarkably, the normally high taurine contents of Müller cells collapse, likely due to osmoregulatory stress [45]. Immediately after light exposure, the outer nuclear layer and external limiting membrane are severely distorted (Figure 4 and Figure 5), with clear hypertrophy of Müller cells. The distortion may represent fluid transport dysregulation. Later in LIRD, fluid channels appear only in LD and decimated zones, implying that the normal transretinal flow of water has been abrogated. Even so, Müller cells seem resilient, as glutamate and taurine levels normalize in survivor zones. Remodeled Müller cells of phase 3 retina show a massive glutamine increase throughout the LD zone (Figure 6). This is not explicable in terms of glutamate overflow from neurons and no amount of endogenous or pharmacologically-induced activity can elevate Müller cell glutamine levels [27,46,47]. The mechanisms for persistent glutamine elevation likely arise from alterations in the normal Müller cell glutamate-glutamine cycle. Extracellular glutamate transported into Müller cells by EAAT1 [44] does not accumulate above 0.1–0.3 mM due the high activity of GS [28,30,31,48]. Normal glutamine levels in Müller cells are on the order of 1 mM [28,30,31] and never reach the >10 mM levels of Müller cells in the LD zone. Further, normal Müller cells use the electrogenic cationic amino acid transporter SN1 (SLC38A3) to export glutamine for neuronal accumulation [49]. LIRD Müller cells are clearly different and mimic astrocytes of central nervous system (CNS) gliomas by expressing increased glutamine content. In gliomas, increased SN1 expression is thought to mediate the accumulation [50], though we don’t know why SN1 switches from export to import. Thus, LIRD Müller cells might transform to net consumers rather than producers of glutamine. This is quite different from the retinal detachment models where GS [51] and glutamine levels [43] drop in parallel. Brain astrocytes do not show major losses in GS even while upregulating glial fibrillary acidic protein (GFAP) in brain trauma [52]. We have not yet examined GS expression in LIRD, but our data predict consistent expression. As Müller cells emigrate from the retina to the choroid, the persistence of the glutamine signal is consistent with the idea that they are transformed and no longer function to serve retinal neurons.

Complex fluctuations in taurine levels as Müller cells transit from normal → remodeled → emigrant cells suggest variations in osmotic stress, oxidative stress and/or taurine availability as mechanisms. Taurine accumulation by Müller cells is mediated by the taurine transporter TAUT [53], and perhaps by the Müller cell GABA transporter GAT3 [54]. TAUT has low retinal expression [55]), is thought to be a key element of glial volume regulation in response to osmotic challenge. Müller cells are also responsible for transretinal water flow through aquaporin-4 (AQP4) channels [56], which puts further demands on volume regulation. Ischemic challenge, such as might be experienced in choriocapillaris atresia, has been shown to upregulate AQP1 in Müller cells as well [57]. In addition, the likely compromise in fluid transport in LIRD zones may account for the formation of amine-rich fluid voids or channels in remnant LIRD retina. Upon re-evaluating our large collection of rodent degeneration models as well as human RP samples, we note that these channels are common and are evident in the histologic analyses of other LIRD experiments [58]. Despite lingering controversy over the actual synthetic sources of taurine and the direction of net taurine transport, most authors agree that systemic taurine is required for photoreceptor survival [59]. The taut^−/−^ mouse [60] develops total photoreceptor degeneration over a year [61] and systemic TAUT inhibitors induce photoreceptor degeneration [62]. A recent quantitative re-evaluation of taurine transport by Hillenkamp et al. [63] convincingly argues that the net direction of RPE mediated taurine transport is from choroid to retina, a direction set by the plasma → subretinal space gradient. Little attention has been payed to the role of Müller cells in taurine transport, but recent work by Tomi et al. [53] also shows that the net taurine transport is toward the retina at the inner blood-retina-barrier (BRB) of the neural retina. It appears that taurine is still available in the LIRD zone, as bipolar cells maintain quite high taurine contents. This makes the decline of taurine in Müller cells all the more surprising. As Müller cells in survivor retina less than 0.1 mm from the LIRD zone have normal τ and Q signals, the content defects in LIRD Müller cell function appear to be cell autonomous. The eventual loss of taurine signals in emigrant cells suggests low bioavailability of taurine in the choroid.

Arginine and CRALBP traces are very similar. CRALBP has long been known as a marker for retinal Müller cells [64] but the role of glia in mammalian retinoid biochemistry is unclear. In LIRD, light stress triggers a rapid increase in CRALBP expression, especially in distal glial processes. This resolves to normal levels in survivor retina, which presumably maintains a normal RPE-photoreceptor retinoid cycle. However, in remodeled LIRD zones that lack both photoreceptors and RPE, Müller cell CRALBP levels reach new heights (millimolar by our estimates), only to collapse upon transformation to emigrant cells. Persistent expression of CRALBP in phase 3 remodeling implies continued availability of retinoids in the neural retina. Similarly retinoid transport and processing persists in animal models of RP, with the novel accumulation of retinoic acid [65], a key activator of developmental [66,67] and adult neurogenesis [68,69] and a potent modulator of neurite extension [70]. In fact, retinoic acid for adult neurogenesis is produced by CNS astrocytes [69]. If the appropriate complexes of retinoid receptors and response elements are present in survivor neurons, elaboration of retinoic acid by Müller cells or other survivor cells may be the global mechanism for de novo neuritogenesis in remodeling, offering the potential for pharmacologic control of remodeling. It is provocative that we have detected elevated expression of cellular retinoic acid binding proteins 1 and 2 (CRABP1/2) in mouse retina after light damage (unpublished data). Further, expression of retinoic acid itself is upregulated in primate models of enhanced postnatal eye growth [71] and excess retinoic acid is associated with a broad spectrum of CNS wiring anomalies [72].

Arginine levels closely track CRALBP expression. While it is common to associate arginine with NO production, other likely more constitutive roles exist for high levels of arginine in cells: regulation of protein synthesis and proliferation. Via arginase, arginine is a major source of cellular ornithine and ultimately polyamines such as spermidine [73]. Polyamines are non-covalent cationic partners [74] for ribosomal stabilization during translation [74,75] and are essential in cell growth and proliferation [76,77], especially in immune cells such as macrophages [78] and lymphocytes [79]. The increase in overall Müller cell mass by hypertrophy [1] suggests a major global cytoskeletal expansion (e.g., enhanced GFAP expression), likely requiring canonical arginine-based protein synthesis regulation shared by prokaryotes and eukaryotes [73,76]. Finally, the complete collapse of CRALBP, arginine and taurine signals as Müller cells become fusiform motile cells is remarkable and its precipitating mechanisms are unknown.

**Table 3:** Percent of eyes in each group displaying remodeling attributes after light exposure.

**Attribute**	**Group**					
	**control**	**pLX 0**	**pLX 14**	**pLX 60**	**pLX 120**	**pLX 240**
Rod D increase	0	100	100	78	0	0
MC R increase	0	100	ND	100	100	100
CRALBP increase	0	100	ND	100	100	100
Misplaced opsin	0	100	ND	67	0	0
Buckle	0	0	31	22	0	0
RPE Q loss	0	0	92	100	100	100
RPE loss	0	0	100	100	100	100
CC loss	0	0	100	100	100	100
MC Q increase	0	0	100	100	100	100
MC t decrease	0	0	100	100	100	100
MC seal	0	0	100	100	100	100
Shortened OS	0	100	100	100	100	90
Microneuromas	0	0	0	100	67	70
Neuronal death	0	0	0	0	17	100
Migration	0	0	0	33	67	20
Emigration	0	0	15	55	60	80
Channels	0	0	38	77	67	70
Rupture	0	0	15	11	33	70
Decimation	0	0	0	22	33	70
MC Q decrease	0	0	0	33	33	80

This table summarizes frequency of twenty discrete remodeling attributes (rows) versus light exposure (columns). Each entry indicates the percent of samples drawn from Table 1 expressing a given remodeling attribute. The five rows show early remodeling attributes, the next seven show persistent remodeling features, and the last eight summarize the occurrence of extreme remodeling features.

### Light-induced retinal damage and age-related macular degenerations

The LIRD model differs from RP-like disorders and more closely resembles the late stages of atrophic AMD. LIRD is initially progressive, but the border between survivor retina and the LIRD margin stabilizes even though continued cell emigration alters the core of the LIRD region. The RPE also manifests very strong changes in metabolite profile including near complete loss of τ and Q signals in stressed regions, even at pLX 0 and a complete ablation of both the RPE and choriocapillaris in zones of LIRD lacking photoreceptors by pLX 14. The process is so fast that it is difficult to determine which cells are the initial fatalities. One structural clue lies in retinal buckling at LIRD margins early in degeneration. As indicated in Figure 6, buckles occur between apparently normal and LIRD retina. Buckles likely represent regions where photoreceptor outer segments continue to lengthen beneath small patches of RPE cells that have lost phagocytic capacity. It is also possible that the appropriate engulfment signals on photoreceptors are also compromised [80]. Even so, RPE cells may succumb to the overload of cyto- and genotoxic oxidative species generated within the RPE, followed by photoreceptor cell loss. It is even possible that the thin, small volume endothelia of the choriocapillaris are affected early. In any event, not only is the BRB ablated in light damage, but the capillary bed that serves the outer retina appears to have undergone atresia. Tanito et al. [58] recently visualized loss of perfusion in the LIRD rat after acute exposure and their histologic findings match ours closely. By all measures, the loss of glutamine in the RPE suggests a massive failure of transport and metabolism and is the earliest concrete sign of irreversible stress in LIRD.

The most dramatic feature of the LIRD phenotype is the escape of transformed retinal neurons into the choroid. First described by Sullivan, Penfold and Pow [81] in the aged “ambient” LIRD rat, it is clear that this is far more than a minor pathology, for it implies that the Müller cell seal is not stable in the long term. We have also observed glial and neuronal emigration in human RP as well (unpublished data) and there is evidence that it may occur in human AMD [82,83]. The emigration appears to be led by Müller cells, with all classes of neurons following. Even with ultrastructural analyses we have not been able to ascertain whether Müller cell transformation to a fusiform shape precedes or follows emigration. We have no clues as to the molecular mechanism initiating the process., Though the shapes of retinal neurons clearly alter during emigration, they preserve their essential GABAergic, glycinergic, or glutamatergic molecular profiles. The emigration process we describe here is much more extensive than the rather orderly extension of Müller cells processes into the choroid in the ambient light aging model. By pLX 240, large expanses of superior retina have been altered by emigration, with little left behind except the processes of survivor amacrine cells and what appear to be the protein skeletons of dead neurons.

AMD, characterized by focal degeneration of central retina, is a leading cause of irreversible blindness [28]. At present, there are at least eight mouse models of human AMD, each manifesting a different phenotype. In most instances, the aberrant phenotypes are slow and mild, generally effecting a focal RPE defect without frank photoreceptor loss. While these models do allow exploration of early intervention strategies for either causal or risk-associated gene products, they are so slow that they provide little progression data. In this context, we show that the LIRD rodent model produces a fast, spatially delimited central RPE-choriocapillaris ablation and is a late-stage AMD mimic. We do not argue that LIRD in any way mimics the provenance of AMD, but that its phase II/III profile represents a retinal loss phenotype potentially similar to atrophic AMD where remnant vision is geographically complex and can rapidly vanish. The LIRD model, which is useful in its own right as a system in which to explore neuroprotection by a vast range of agents (e.g., [84-87]), may be a good match to the advanced features of late atrophic AMD. LIRD is regional, with very sharp transitions between affected and survivor retina, similar to AMD and very unlike most RP-like disorders. More specifically, even though RP can present mixed survivor and degenerating retina, the transition zones are between phase I and II retina or phase II and III retina (see Rayborn et al. [88]). In contrast, both LIRD and geographic atrophy present distinctive phase I/phase III borders. This transition extends into the choriocapillaris in LIRD and AMD, but generally not in RP. Vascular perfusion is directly impaired in LIRD [58]. The LIRD defect initiates in a “sensitive” region, superior retina in rodents [89] and human AMD is biased toward the macula. The choroidal vasculature of the dorsal (superior) hemisphere of rat eye is sparser than the ventral, which is served by the dense inferior branch arteriole [90] and the relative resistance of the latter to LIRD is possibly due to greater perfusion. Similarly, the macular vasculature appears to have a lower perfusion rate, which may be associated with preferential though not absolute risk. More concretely, geographic atrophy in the macula is explicitly accompanied by severe degeneration of the choriocapillaris in affected zones, but not in survivor retina [91]. The LIRD defect is initiated at least at the RPE, the BRB is compromised and there is direct commerce between the choroid and the subretinal space. Loss of RPE function will cause photoreceptor loss in both AMD and LIRD. Both LIRD and AMD are associated with oxidative stress in the outer retina, and numerous forms of antioxidant-based neuroprotection for acute LIRD (e.g., [85-87,92] have parallels in studies showing endogenous antioxidant carotenoids to be protective in AMD [20].

Finally, the similar patterning of survivor and decimated retina in LIRD and human geographic atrophy (compare Figure 12 and Figure 16) is striking. It is unfortunate that we have no access to rapidly fixed late atrophic AMD samples as the small molecule signatures in the human retina shown in Figure 16 have been lost. Even so, the retention of CRALBP signals shows the retinal decimation to be substantial. More explicitly, RP-like diseases in humans and rodents do not manifest decimated retina or emigration, and while there are significant cell losses in some forms of RP (e.g., adRP [1]) all survivor cells in RP-like diseases express robust small molecule signatures [1,5]. Conversely, spatially discrete retinal decimation is a hallmark of both LIRD and AMD. Does emigration occur in AMD? We do not yet know and see little evidence of it in this one sample. However, it is likely that emigrant neurons eventually die and, after months or years in the choroid, neuron- or glia-specific markers will disappear from survivor emigrant cells, just as CRALBP and arginine expression disappear from emigrant Müller cells. Many features of the LIRD model (morphological and molecular revision of photoreceptors, Müller cells, the RPE, Brüch’s Membrane and choriocapillaris) may prove to be effective surrogates for the study of late stage AMD. In parallel, CMP and our emerging panel of small molecule stress markers may also be a powerful way to characterize early glial, RPE and neuronal transformations during periods of drusen formation or RPE-choriocapillaris immune stress in rodent models of early AMD [92-97].

### Conclusions

**Figure 19:**
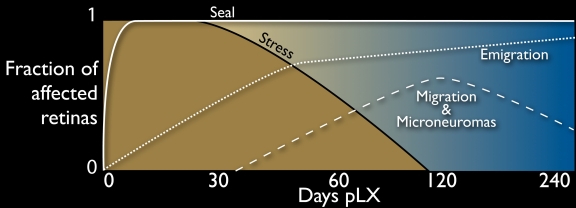
Temporal profile of remodeling in light induced retinal damage. A schematic of the fraction of retinas displaying remodeling attributes (see Table 3) displayed on an exponential time scale. Immediately after light exposure, all retinas show massive stress signals, but by pLX 120 these are no longer evident. Müller cell seals form rapidly after light exposure and persist. Breakdown of the seal can be detected by pLX 14 and increases with time. Classic remodeling phenomena such as migration and microneuromas are evident by pLX60 but decline after emigration and retinal decimation become dominant.

Environmental or inherited insults that kill photoreceptors are sensory deafferentations with the potential to trigger neural remodeling including neuronal loss, growth of new neurites, formation of new synapses, and even somatic migration. LIRD is a fast, effective trigger of large-scale remodeling (perhaps due to the high temporal coherence of the insult) that replicates and then progresses past phase III remodeling to ultimately decimate the neural retina (Figure 19). LIRD will enable detailed study of circuitry defects emergent from remodeling [5,6]. Moreover, the LIRD model presents derangements of cellular functions ranging from energetics (aspartate stress), osmoregulation (taurine dysregulation), retinoid processing (CRALBP expression fluctuations), global protein translation modulation (arginine upregulation), to glioma-like changes in glutamine metabolism. Collectively, these data demonstrate that LIRD models exhibit pathological processes observed in other retinal degenerative diseases and a variety of general neurodegenerations and neoplastic transformations. The LIRD model is a highly practical model for mimicking degenerative retinal disease without complicating developmental side-effects. Finally, the LIRD model is the *only* available model exhibiting all of the hallmarks of AMD including choroidal ablation, RPE ablation, photoreceptor cell death and retinal decimation.
